# Simulations and Experiments on the Micro-Milling Process of a Thin-Walled Structure of Al6061-T6

**DOI:** 10.3390/ma15103568

**Published:** 2022-05-17

**Authors:** Qi Sun, Jianzhong Zhou, Pengfei Li

**Affiliations:** School of Mechanical Engineering, Jiangsu University, Zhenjiang 212013, China; zhoujz@ujs.edu.cn (J.Z.); 1000005227@ujs.edu.cn (P.L.)

**Keywords:** simulation, micro-milling, thin-walled, Al6061-T6, micro deformation

## Abstract

Aluminum alloy (Al6061-T6) is an alloy with strong corrosion resistance, excellent disassembly, and moderate strength, which is widely used in the fields of construction, automobile, shipping, and aerospace manufacturing. Researching on the influence of machining precision and surface quality on the micro-milling process of thin-walled structures of Al6061 is highly significant. Combined with the two simulations (DEFORM-3D simulation and interactive finite element numerical simulation (FEM)) and milling experimental verification, the deformations, errors, and surface quality of milling thin-walled Al6061 were analyzed. The simulations and experimental results show that the deformation of milling a micro thin-walled structure was caused by the vertical stiffness of the thin-walled structure and the cutting force. Surface micromorphology further characterized and showed a poorer quality area, top burr, and concave defects, which directly affect machining quality. It is necessary to improve the surface quality, reduce the surface defects, and increase the stiffness at the top of thin-walled structures in future work.

## 1. Introduction

Milling thin-walled structures has been widely used in numerous fields, such as aerospace, microelectronics, and medical applications [1,2]. Therefore, the milling process was studied using micro thin-walled structures of Al6061-T6. There are many problems in milling micro thin-walled structures, such as deformations and bending in particular. In 2007, Wan et al. [3,4] contrasted three methods to select the best feed per tooth and depth of cut simultaneously: (1) attempting to obtain the largest feed per tooth ignoring tolerance; (2) obtaining the suitable cutting parameters by solving the linear equation problem; (3) using the method of symmetric error compensation. Finally, surface errors were measured using the three methods. In 2008, the deformation model was conducted using ANSYS [5], which can be used to analyze the deformation effect of different forces, tool positions, and thicknesses of thin-walled structures. To analyze the deformations of the thin-walled structures of Ti6AL4V, Gang et al. [6] established three-dimensional (3D) finite element models of a helical end milling cutter and cantilever beam. Experiments were conducted to verify the simulation results under the same conditions. Ning et al. [7] used the finite element method (FEM) and control strategy to calculate the deformation of typical thin-walled structures. The FEM model of multi-frame components was established to predict deformations under different machining conditions [8,9].

Some scholars have machined thin-walled structures with a high aspect ratio with different thicknesses and materials [10,11]. Kou et al. [12] found that a U-shape could reduce the deformation. A low-melting point alloy was used to balance the radial cutting force and improve the rigidity of the thin-walled workpiece, and a micro thin-walled structure of 15 μm thickness was machined to verify the method. Sridhar et al. [13] studied the influence of tool diameter and found that the deformations were too large with an incorrect tool. The experiments were conducted with the same feed speed, spindle speed, cutting depth, and cutting tool size. Guo et al. [14] analyzed the force signal in the time and frequency domains and compared the effects of different machining parameters. Conditions, such as the thickness of the thin-walled structure, milling method, and edge radius caused by machining wear were analyzed [15]. In particular, the relationship between the machining quality and cutting force was studied. The coupled dynamic response of a thin-walled structure and tool, which was helpful in understanding the influence of the dynamic effect and design of passive damping, was studied [16,17]. Finally, Rai et al. [18] systematically introduced the FEM.

Some phenomena, which could not be seen easily in experimental machining, could be observed by FEM simulation [19,20]. A software package for finite element analysis was developed by Ratchev et al. [21], who researched the simulation deformations. Cheng et al. [22] simulated the milling process of titanium alloys based on the characteristics of the deformations and cutting forces. Huang et al. [23] studied the monolithic component deformation combined with FEM simulations and experiments, and then attempted to eliminate the residual stress on the surface. Li et al. [24] explored the relationship between cutting parameters and cutting force using FEM. The influence of cutting heat on the machining quality was analyzed using a simulation-aided machining method [25]. Thepsonthi et al. [26] simulated the cutting force and milling temperature, in contrast to the results of the 2D and 3D simulations. They found that 3D simulation was better but required more time. Titanium alloy millings with different coating tools were simulated by Özel et al. [27]. Arrazola et al. [28] pointed out that DEFORM-3D simulation could be used to predict the surface roughness of a thin-walled structure.

Summarizing the research above, majority of the studies are focused on the milling process of conventional thin-walled structures with thicknesses of several millimeters. However, until now, there have been few systematic investigations concerning micro thin-walled structures that play an important role in many fields. Therefore, an attempt was made in this study to establish a deformation prediction model using FEM. First, the cutting force and surface location errors were analyzed. A deformation prediction model using iterative FEM was established. Moreover, a DEFORM-3D simulation was conducted to analyze both the thickness error and surface topography. Finally, the deformation, machining error, and surface machining quality are discussed.

## 2. Modeling of a Micro Thin-Walled Structure by FEM

### 2.1. Modeling of the Cutting Force of Micro Thin-Walled Structures

**Figure 1:**
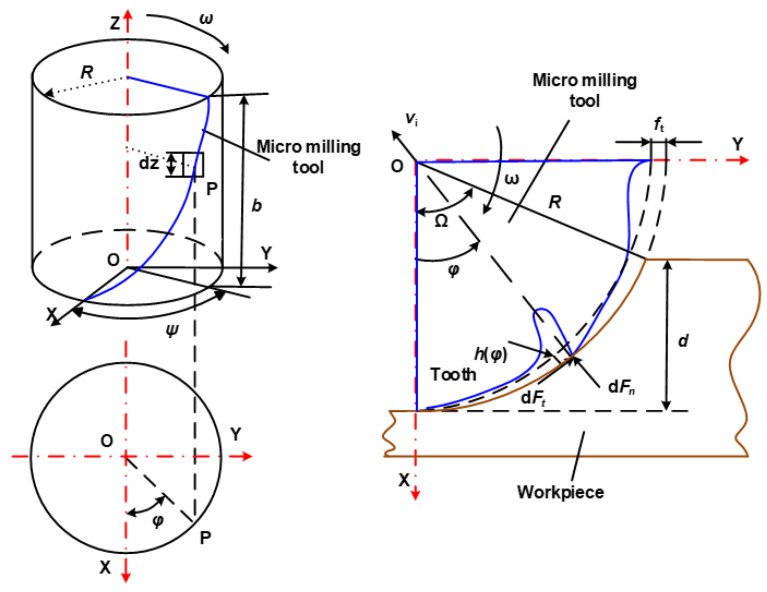
Different cutting model of peripheral milling [29].

The micro-milling tool can be divided into many pieces along the z-direction, as shown in Figure 1. Within each slice, a model of one cutting edge can be established, and the radial and tangential cutting forces on the rake face can be obtained by the model. The total cutting forces of the end-milling tool can be calculated by integration.

(1) $$dF_{t}\;=K_{t}h\left(\varphi \right)dz,\left(0<\varphi <\psi \right)$$

(2) $$dF_{n}\;=K_{n}h\left(\varphi \right)dz,\left(0<\varphi <\psi \right)$$

where 

d*F*_t_—Tangential cutting force of each slice,

d*F*_n_—Radial cutting force of each slice,

*K*_t_—Coefficient of the tangential cutting force,

*K*_n_—Coefficient of the radial cutting force,

*h*—Cutting thickness at the cutting angle of *φ*,

dz—Discrete axial cutting depth,

*ψ*—Maximum cutting angle,

*φ*—Cutting angle at the present cutting state.

The actual path of any point at the milling tool was cyclic during the milling process. The linear length was small relative to the tool radius. The path of the rotational speed can be seen as a series of circles, therefore, the cutting thickness and feed per tooth *f*_t_ can be expressed almost as

(3) $$h=f_{t}\sin\left(\varphi \right)$$

where *φ*—Rotational angle,

*f*_t_ —Offset per tooth, which can be obtained by the linear feed speed *f*, spindle speed Ω, and the number of cutter edges *N*_t_.

Here, the units are μm/tooth for the feed per tooth, mm·s^−1^ for the linear feed rate, rpm for spindle speed, and teeth/rev for the number of teeth.

(4) $$f_{t}=\frac{60f}{\Omega N_{t}}\times 1000$$

The relationship among the coefficients of the cutting force, edge radius, and thickness can be obtained by Jin [30].

(5) $$K_{t}(h,r)=\alpha_{t}h^{d_{t}}+\beta_{t}h^{p_{t}}r^{q_{t}}$$

(6) $$K_{n}(h,r)=\alpha_{n}h^{d_{n}}+\beta_{n}h^{p_{n}}r^{q_{n}}$$

Here, *α*, *β*, *d*, *p*, and *q* are the coefficients (these parameters are empirical values obtained from previous extensive experiments and simulation work) relative to the materials, and thus the cutting force coefficient can be obtained using mathematical calculation software. Subsequently, the material coefficients were converted to Al6061-T6, and the coefficient of cutting forces was calculated, as shown in Table 1.

Now that the total radial cutting force and tangential cutting force can be obtained by integration, the forces in the *x* and *y* directions can be obtained. The force in the –*y* direction was considered mainly because it can directly affect the deformation of the micro thin-walled structure.

(7) $$F_{x}=F_{t}\cos\left(\varphi \right)+F_{n}\sin\left(\varphi \right)$$

(8) $$F_{y}=F_{t}\cos\left(\varphi \right)-F_{n}\sin\left(\varphi \right)$$

The tangential and normal force components can now be substituted in Equations (9) and (10).

(9) $$F_{x}=K_{t}bf_{t}\sin\left(\varphi \right)\cos\left(\varphi \right)+K_{n}bf_{t}\sin\left(\varphi \right)\cos\left(\varphi \right)$$

(10) $$F_{y}=K_{t}bf_{t}\sin\left(\varphi \right)\cos\left(\varphi \right)-K_{n}bf_{t}\sin\left(\varphi \right)\cos\left(\varphi \right)$$

### 2.2. Analysis of Surface Location Error

**Figure 2:**
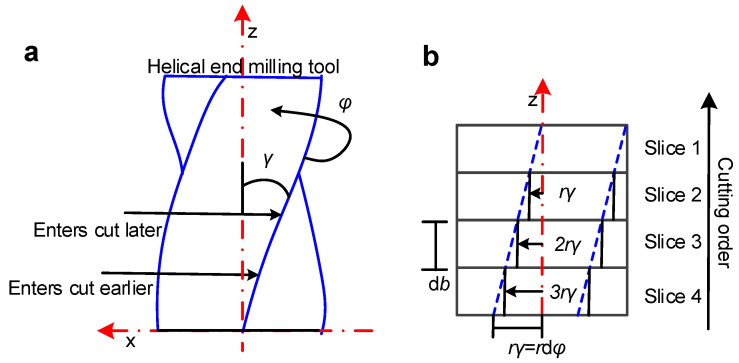
(**a**) Helical end milling tool geometry causing cutting edge time delay; (**b**) discretized version of unrolled helical end milling tool geometry.

The effect of the helical end milling tool geometry is that the entire cutting edge cannot be cut at the same instant. The lower part of the cutting edge cuts first followed by the upper part. The geometry of the helical end-milling tool is shown in Figure 2.

The surface location error along the tool is continuous, and it is cyclic in the z direction if the variety of stiffness of the workpiece and milling tool are ignored. Similar to the calculation of the cutting forces, it was also divided along the z direction. Each slice (whose thickness was d*b*) can be seen as an individual straight end milling tool.

The surface location error can vary with the change in height, and it is always along the cutting edge of the helical end milling tool. Here, the error in the y-direction is mainly considered, and the delay angle *χ* along the tool direction can be expressed as

(11) $$\chi =\frac{2\left|z\right|\tan(\gamma )}{d}$$

where $\left|z\right|$—Height from bottom to the calculative position,

***d***—Diameter of milling tool,

***γ***—helix angle of the milling tool.

The corresponding time delay ***t*** is

(12) $$t=\frac{60\cdot \chi }{2\pi \cdot \Omega }$$

where Ω—Spindle speed.

The coefficients of the Fourier transform can be obtained based on the parameters of the milling tool and cutting condition, and the frequency response function (FRF) in the y direction can be expressed as

(13) $$FRF_{y}=\frac{1}{k_{y}(\frac{{\left(j\omega \right)}^{2}}{\omega_{ny}{}^{2}}+\frac{2\xi_{y}\omega }{\omega_{ny}}j+1)}$$

where k_y_ —Equivalent stiffness of the tool system,

*ω*_ny_ and *ζy-modal* parameters of the system.

**Figure 3:**
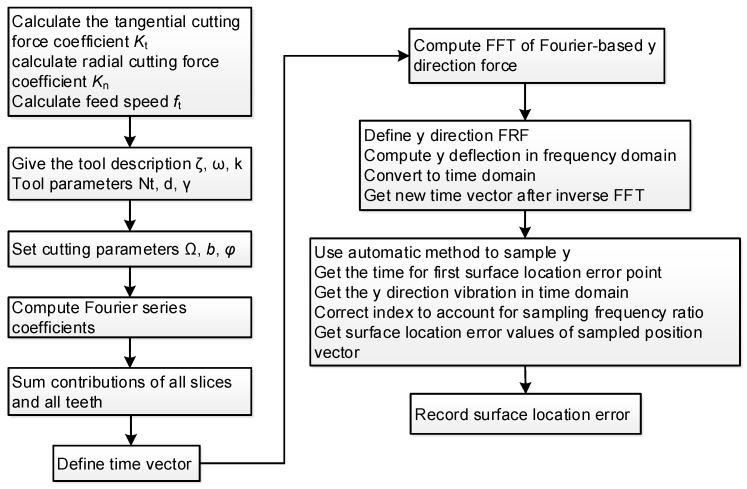
Calculation process of surface location error variation with axial location.

In the calculation of mathematical calculation software, we should sample the nodes, calculate the tool pass time, and time increment, and calculate the surface location error. The entire process is illustrated in Figure 3.

### 2.3. Error Prediction of Cutting Deformation and Error

**Figure 4:**
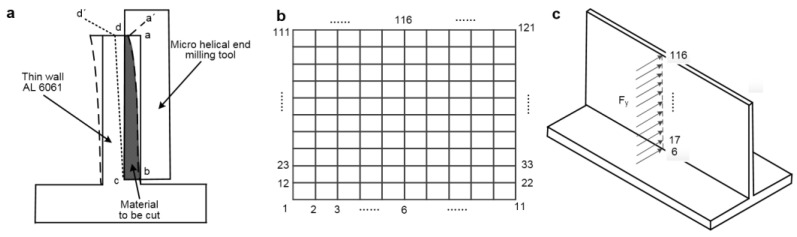
Micro thin-walled milling model: (**a**) error analysis of the micro thin-walled structure; (**b**) distribution of calculation points; (**c**) distribution of milling force.

The micro thin-walled milling model is shown in Figure 4. In Figure 4a, the area abcd should be theoretically cut. However, only area a′bcd was cut off because of the low stiffness and deformation caused by the cutting force. In conclusion, points a and d were pushed to points a′ and d′, and the area of a′bcd was cut. When there is no contact between the tool and workpiece, the micro thin wall recovers slightly, and deformation and error appear.

If the deformations of the entire wall structure can be obtained, the residual part can be cut using the digital control compensation method. However, it is important to obtain the machining error values and reduce the error caused by the micro-milling tool as much as possible under the given cutting parameters.

There are 100 units in the micro thin-walled model (Figure 4b), whose material properties and element sizes are similar. The cutting force was scattered on these nodes: 17, 28, 39, 50, 61, 72, 83, 94, 105, and 116 (Figure 4c). The stiffness matrix [29] for each unit is expressed as

(14) $$k=\int B^{T}DBz^{2}dV$$

where ***B*** is the strain matrix of each unit, *z* is the thickness of the micro thin-walled structure, and ***D*** is the elastic matrix of each unit.

The total stiffness matrix of the entire thin-walled structure ***K*** can be obtained by superposing the stiffness matrix of each unit. The cutting force matrix ***F*** can be obtained based on the node numbers and location of the force. After the calculation, the deformations of each node are obtained as follows:

(15) $$\delta_{363\times 1}={\left(K_{363\times 363}\right)}^{-1}F_{363\times 1}$$

**Figure 5:**
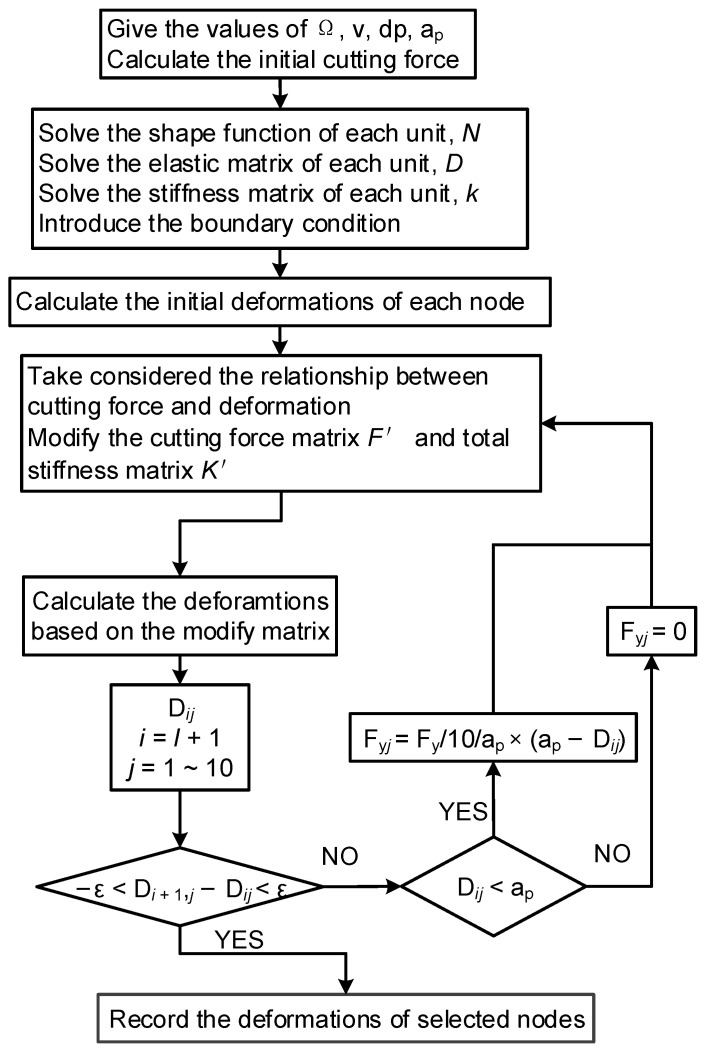
Analyzed flow chart of micro thin-walled structure deformation.

Because the nodes from 1 to 11 are fixed, the deformations of these nodes are zero. The cutting force and deformations affect each other during the machining process. Therefore, the interrelationship between force and deformation is considered by the iterative FEM. In this method, the total stiffness matrix ***K*’** and cutting force matrix ***F*’** are modified after each cyclic calculation based on the deformations of each node. The entire procedure is illustrated in Figure 5.

## 3. DEFORM-3D Modeling and Simulation

### 3.1. Three-Dimensional Modeling of Milling Micro Thin-Walled Structure

**Figure 6:**
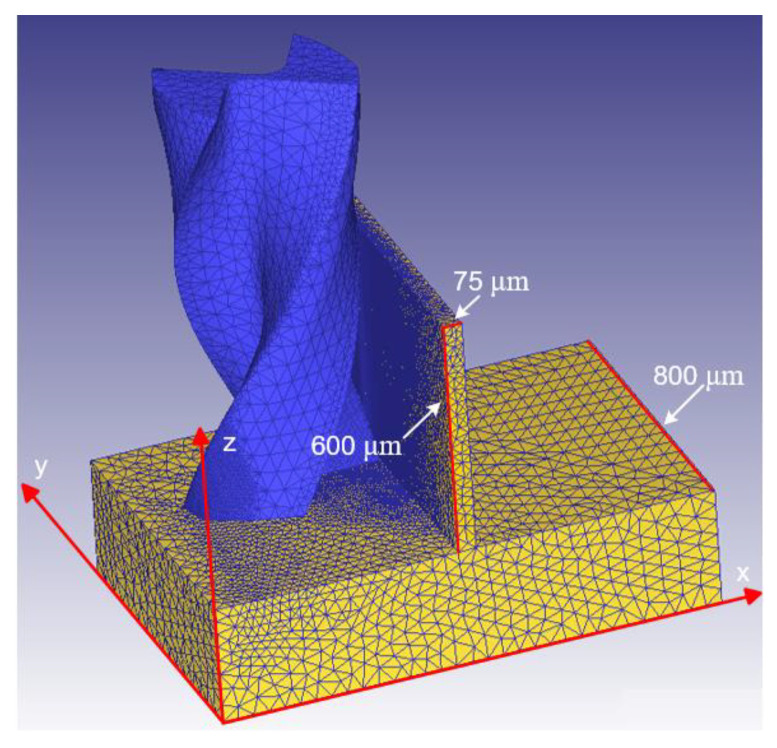
Geometric model of the tool and micro thin-walled structure.

The milling of thin micro-wall structures using a diamond-coated micro-milling cutter was studied. The tool and micro thin-walled structures were modeled by real measurements and then modeled using 3D software, as shown in Figure 6. The geometric models of the tool and workpiece were based on the parametric modeling of the 3D drawing software, and then imported into the deform software. The machining material was used an elastic model, and the cutter was used a rigid model. The thickness, height, and length of the thin micro wall were 75, 600 and 800 μm, respectively. Tetrahedral mesh was used in the model. The number of the elements was about 100,000. 1 week (7 × 24 h) was taken to calculate. The axial cutting depth, rotation direction, and feed speed were h, clockwise, and f, respectively. In the coordinate system, the center of the milling cutter is the origin of the coordinate, the feed direction for the –y direction, the tool axial direction for the z direction, and the x axis is perpendicular to the plane of the y and z axes. In the milling simulation, the starting point of the cutting angle was zero. The entire workpiece was fixed, except for the part of the thin-walled structure. The feed speed was 0.5 mm·s^−1^ along the –y direction, the axial cutting depth was 600 μm, and the spindle speed was 15,000 rpm.

### 3.2. Material Properties

The constitutive model of the Al6061-T6 work material is used as given by Johnson-Cook [31]:

(16) $$\sigma =\left(A+B\varepsilon^{n}\right)\left(1+C\ln\frac{\dot{\varepsilon }}{{\dot{\varepsilon }}_{0}}\right)\left[1-{\left(\frac{T-T_{r}}{T_{m}-T_{r}}\right)}^{m}\right]$$

where

ε—Equivalent plastic strain,

$\dot{\varepsilon }$—Equivalent plastic strain rates

${\dot{\varepsilon }}_{0}$—Reference plastic strain rates

T—Material temperature of cutting zone,

T_r_—Melting temperature,

T_m_—Room temperature,

n—Strain hardening index,

m—Thermal softening index.

A—Yield strength of the material,

B—Strength coefficient of the material,

C—Strain-rate sensitivities of the material.

The material constants of Al6061-T6 were adjusted by Jin [30] and are given in Table 2. The other physical properties of the materials used in the simulation are summarized in Table 3. The geometric parameters of the micro-milling tool are listed in Table 4.

### 3.3. Friction and Heat Transfer

The friction heating phenomenon occurs severely during the milling process. The stress flow properties vary with the increase in temperature of the workpiece material, and this affects the cutting force in the simulation. The contact between the cutter and the workpiece is divided into two types: adhesive contact and sliding contact. Therefore, the corresponding friction types are shearing friction f_s_ and Coulomb friction f_c_; as shown in Equation (17), the friction coefficients of a and b are 0.9 and 0.7, respectively. The values of *a* and *b* are obtained based on processing parameters and a large number of experimental values. To simulate the process of chip production, the contact method was set to adhesive contact, and the contact factor was 0.2.

The unit of the heat transfer coefficient, which is used to simulate the heat transfer phenomenon of the contact area in the milling process, is 10^6^ (W/(K m^2^)). The initial temperature of the cutter and workpiece were both 20 °C, and the value of the heat transfer coefficient was 0.02.

(17) $$\left\{\begin{array}{c} f_{s}=a\times k\left(a\times k>b\times j\right)\\ f_{c}=b\times j\left(a\times k\leq b\times j\right) \end{array}\right.$$

*a*—Friction factor in the adhesive friction zone

*k*—Shear yield stress of workpiece material,

*b*—Friction factor in the sliding friction area,

*j*—Contact stress between cutter and workpiece.

## 4. Experimental Processes

**Figure 7:**
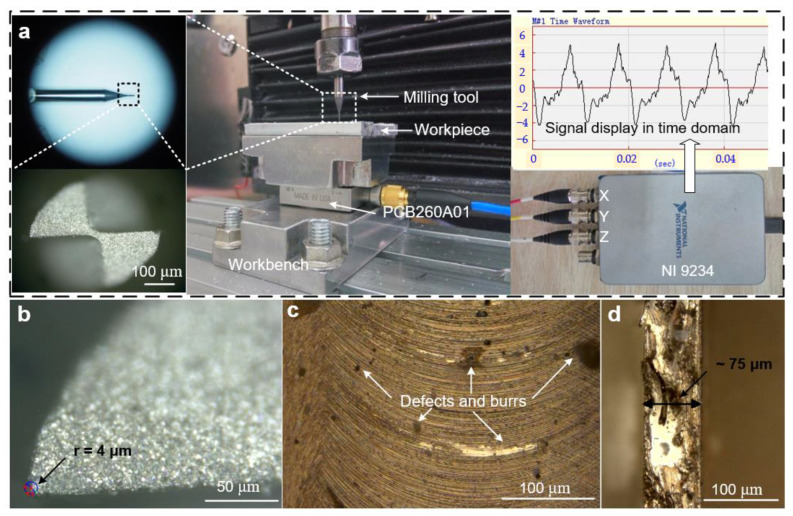
(**a**) Experimental setup and milling force measurement system; (**b**) measure the size of tool-edge radius and observe the tool geometry; (**c**) milled surface morphology and defects; (**d**) measure the thickness of micro thin-walled structure before and after processing from the top view.

The processing equipment used in the experiments is shown in Figure 7a. The maximum speed of the air-cooled electric spindle was 24,000 rpm. A digital microscope can observe the geometry of the micro-milling tool and measure the edge radius. The workpiece was fixed at PCB260A01, the cutting force signal in the cutting process can be collected in real time by NI9234, and finally, the signal is analyzed and shown by the ME’scope. A VHX-1000E digital microscope was used to measure the size of the tool-edge radius (Figure 7b), observe the milled surface and top morphology (Figure 7c,d) of the thin-walled structure after processing, and measure the thickness of the micro thin-wall before and after processing from the top view. It has two lenses with different magnifications, which are 20–200 times and 500–5000 times, respectively. According to the observation results, it can be found that the edge radius of the diamond-coated micro-milling tool is 4 μm approximately, there are still some defects and burr on the milled surface, and the thickness and top burr of the thin-walled structure can be observed.

## 5. Results and Discussion

### 5.1. Deformation of Iterative FEM

In the iterative FEM simulation of deformation, the entire micro thin-walled structure was divided into 100 quadrilateral elements, and there were 121 nodes. In this study, the thickness of the thin-walled structure was 75 μm, and the nominal radial cutting depth was 8 μm. In the calculation process, the cutting force is divided into nodes on the vertical line to approach the actual processing (nodes 17, 28, 39, 50, 61, 72, 83, 94, 105, 116).

**Figure 8:**
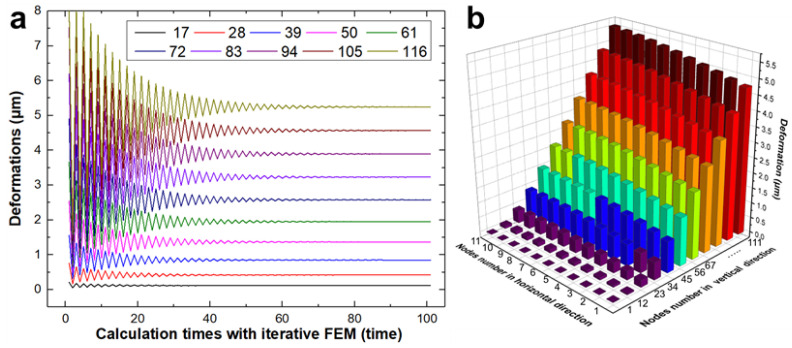
(**a**) Deformations of parts nodes in the iterative calculation process; (**b**) all node deformations in horizontal and vertical direction.

The deformations of the nodes with force were mainly considered. Based on the calculation method in Figure 5, the nominal radial cutting depth was modified, and the real radial cutting depth was obtained according to the deformations of the nodes in the current cycle. The relationship between the deformations of nodes with force and the iterative calculation frequency is shown in Figure 8a.

Figure 8a shows that the deformations fluctuate considerably and are unstable at the beginning of the iterative loop. This illustrates that the maximum deformation is too large without iterative FEM and even larger than the nominal radial cutting depth. The deformation cannot be larger than the radial cutting depth, thus demonstrating the importance of the iterative FEM. After several iterative loops, the deformations of the selected nodes tended to be stable one by one. From top to bottom of the thin-walled structure, the deformations gradually approached zero, and the steady calculation period became shorter. Figure 8b shows the deformations of all the nodes. Deformations increase from the bottom to the top in the horizontal direction, and the maximum deformation, which is approximately 5.5 μm, is located in the top corner. The deformations in the vertical direction are very close because of the small deviations in the calculation of the element stiffness.

### 5.2. Deformations of Deform-3D Simulation and Experiments

**Figure 9:**
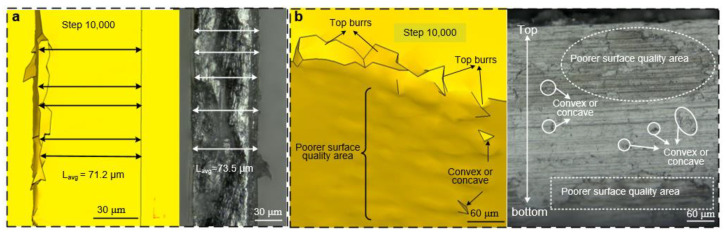
Comparison of milled thin-walled structures between simulation and experiments: (**a**) thicknesses and (**b**) surface quality.

To verify the thickness error of the micro thin-walled structure, experiments and DEFORM-3D simulations were conducted. The results are shown in Figure 9a. The left diagram shows the measurement results of the DEFORM-3D simulation, and the right diagram shows the experimental results. The thicknesses of the five positions were measured randomly, and the average value was considered as the maximum thin-walled thickness after machining. Multiple tests and repeated measures were performed to reduce errors. The results show that the thicknesses of both the experiments and the simulation are larger than the ideal thickness. The average thickness of the DEFORM-3D simulation was 71.2 μm, and the experimental average thickness was 73.5 μm.

It is difficult to measure the surface roughness and position error using a 3D profile-measuring instrument because of the small area of the thin-walled structure. Therefore, in this study, the DEFORM-3D simulation was used to observe the surface quality, and experiments were conducted to observe the surface topography using a VHX-1000E digital microscope, as shown in Figure 9b. The left picture shows the surface topography obtained by simulation. The poorer surface quality area can be seen clearly; the convex or concave areas appear on the front of the thin-walled structure, and there are large burrs on the top of the thin-walled structure. These defects directly affect the machining quality. The right picture shows the elevation view of the thin micro wall after machining. There are two poorer surface quality areas and several convex or concave areas located on the entire surface, verifying the results of the DEFORM-3D simulation. In the real experiments, there are many burrs, especially top burrs, as shown in the right picture of Figure 9b. The surface quality is very poor at the top of the thin-walled structure, and this may be because the top burr or sharp high-temperature chips remain and scratch the top side of the thin-walled structure. Therefore, it will be an important project to reduce the top burr and separate chips in the future.

Considering the above three analysis methods, including iterative FEM, DEFORM-3D simulation, and experiments, the different thicknesses after machining are shown in Table 5, where the nominal radial cutting depth is 8 μm and the thickness before the final machining is 75 μm. The results show that the thicknesses are all larger than the ideal thickness of 67 μm. Since the material is not rigid, the milling force causes the thin-walled structure to bend slightly, so the actual milling width is less than ideal width. The error range between obtained results and ideal values is about 7.5%. The result is basically the same as the research results of Jia [33], even smaller than their error range. This phenomenon illustrates that there is indeed a decrease in material stock removal when milling micro thin-walled structures. The experimental results are slightly larger than those of the DEFORM-3D simulation and iterative simulation, which may be because of cutter run-out. Sometimes, the tool is too far away from the workpiece, and sometimes the radial cutting depth is too large, causing it to bend and reduce the amount of material removal.

**Figure 10:**
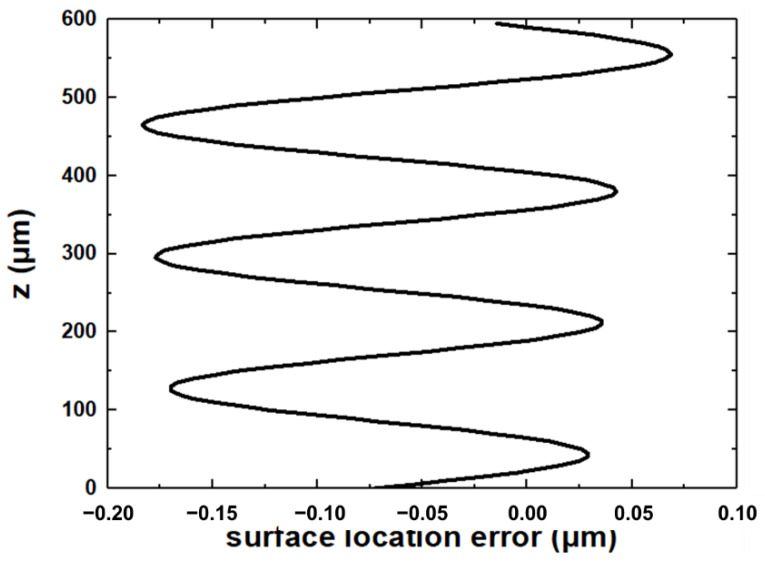
Surface location error variation with axial length.

The surface location error can be obtained based on Figure 3. Here, the axial cutting depth *b* is 600 μm, the spindle speed Ω is 15,000 rpm, and the helix angle *γ* is 30°. According to the modal testing and ME’scope software analysis, the equivalent stiffness k_y_ is 0.01, and the modal parameters of the system *ω*_ny_ and *ζ*_y_ are 4035 Hz and 2.1425 × 10^6^ N·m^−1^, respectively. Therefore, the relationship between the surface location error and the axial height is shown in Figure 10. In the micro thin-walled structure, the surface location error is large due to the low stiffness at the top of the micro thin-walled structure. On the contrary, the micro thin-walled stiffness at the bottom is higher and the deformation is smaller, so the surface location error is small. The surface location error increased slightly with decreasing micro thin-walled stiffness along the vertical direction. There are several methods to improve the milling quality, including reducing deformation, optimizing processing parameters, controlling chatter, and increasing support.

## 6. Conclusions

The numerical iterative FEM prediction model and Deform-3D simulation model of micro-milling process are established in order to predict the thickness of a Al6061-T6 thin-walled structure. By comparing the results of simulations and experiments, the conclusions were obtained as follows.

1. The thickness of the thin-walled structure obtained by the iterative FEM simulation, DEFORM-3D simulation and experiments are all larger than the ideal value, illustrating that there is a deformation in the process of micro-milling processing.
2. The final machining thickness error among the three methods is less than 2.5 μm, providing a reference for calculating the deformation and final thickness of thin-walled structures according to the milling force.
3. Affected by the vertical stiffness, the deformation at the top of the thin-walled structure is smaller than that at the bottom. The weak stiffness at the top of the thin-walled structure leads to a higher surface location error.
4. Some defects (convex or concave poorer surface quality area, and top burr) appears on the thin-walled structure surface, which directly affect the machining quality. Decreasing the deformations and defects is necessary to improve the surface quality and processing accuracy.

## Figures and Tables

**Table 1:** Values of cutting coefficient for Al6061-T6.

Items	Values	Items	Values
*α*_t_	490	*α*_n_	128.8
*d*_t_	–0.351	*d*_n_	–0.3355
*β*_t_	2123	*β*_n_	894.8
*p*_t_	–0.719	*p*_n_	–0.9272
*q*_t_	1.055	*q*_n_	0.9696

**Table 2:** Johnson-Cook model parameters of Al6061-T6 [30].

A (MPa)	B (MPa)	C	N	m	T_m_ (K)	T_r_ (K)	${\dot{\varepsilon }}_{0}$(s^−1^)
324	114	0.011	0.35	1.34	293.15	923.15	1

**Table 3:** Mechanical and thermal properties of Al6061-T6 and tool materials [32].

Density (kg⋅m^−3^)	Elastic Modulus (GPa)	Poisson’s Ratio	Conductivity (W∙m^−1^∙K^−1^)	Specific Heat (J∙kg^−1^∙K^−1^)	Inelastic Heat Fraction
2700	68.9	0.33	167	896	23.6

**Table 4:** Geometric parameters of the micro-milling cutter.

Rake Angle (°)	Relief Angle (°)	Edge Radius (μm)	Cutter Diameter (μm)	Helix Angle (°)
–2	17	4	500	30

**Table 5:** Final machining thickness comparison among different methods.

Methods	DEFORM-3D Simulation	Iterative Simulation	Experiment
Thickness (μm)	71.2	72.5	73.5

## Data Availability

Not applicable.
